# Routine bacterial culture of proximal bone specimens during minor amputation in patients with diabetes-related foot infections has little clinical utility in predicting re-operation or ulcer healing

**DOI:** 10.1186/s13047-022-00563-2

**Published:** 2022-08-20

**Authors:** Kimberly Voon, Uyen G. Vo, Robert Hand, Jonathan Hiew, Jens Carsten Ritter, Emma J. Hamilton, Laurens Manning

**Affiliations:** 1 Multidisciplinary Diabetes Foot Ulcer Service, Fiona Stanley Hospital, Murdoch, Australia; 2 Department of Endocrinology, Fiona Stanley Hospital, Murdoch, Australia; 3 Department of Vascular Surgery, Fiona Stanley Hospital, Murdoch, Australia; 4 Department of Infectious Diseases, Fiona Stanley Hospital, Murdoch, Australia; 5 Department of Podiatry, Fiona Stanley Hospital, Murdoch, Australia; 6 School of Medicine, Curtin University, Bentley, Australia; 7 Medical School, University of Western Australia, Harry Perkins Research Institute, Fiona Stanley Hospital, PO Box 404, Bull Creek, Western Australia 6149 Australia

**Keywords:** Diabetes, Foot, Diabetes-related foot ulcer, Osteomyelitis, Minor amputation, Bone, Swab

## Abstract

**Background:**

Trans-phalangeal and trans-metatarsal amputation, collectively termed ‘minor amputations’ are important procedures for managing infections of diabetes-related foot ulcers (DFU). Following minor amputation, international guidelines recommend a prolonged course of antibiotics if residual infected bone on intra-operative bone samples are identified, but the quality of the evidence underpinning these guidelines is low. In this study, we examined the concordance of microbiological results from proximal bone cultures compared to results from superficial wound swabs in relation to patient outcomes; with the aim of determining the utility of routinely obtaining marginal bone specimens.

**Methods:**

Data was retrospectively collected on 144 individuals who underwent minor amputations for infected DFU at a large Australian tertiary hospital. Concordance was identified for patients with both superficial wound swabs and intra-operative bone samples available. Patient outcomes were monitored up to 6 months post-amputation. The primary outcome was complete healing at 6 months; and secondary outcome measures included further surgery and death. Mann Whitney U testing was performed for bivariate analyses of continuous variables, Chi-Squared testing used for categorical variables and a logistic regression was performed with healing as the dependent variable.

**Results:**

A moderate-high degree of concordance was observed between microbiological samples, with 38/111 (35%) of patients having discordant wound swab and bone sample microbiology. Discordant results were not associated with adverse outcomes (67.2% with concordant results achieved complete healing compared with 68.6% patients with discordant results; *P* = 0.89). Revascularisation during admission (0.37 [0.13–0.96], *P* = 0.04) and amputation of the 5th ray (0.45 [0.21–0.94], *P* = 0.03) were independent risk factors for non-healing.

**Conclusion:**

There was a moderate-high degree of concordance between superficial wound swab results and intra-operative bone sample microbiology in this patient cohort. Discordance was not associated with adverse outcomes. These results suggest there is little clinical utility in routinely collecting proximal bone as an adjunct to routine wound swabs for culture during minor amputation for an infected DFU.

## Introduction

Globally, one person loses a limb every 30 seconds, usually as a direct consequence of an infected diabetes-related foot ulcer (DFU) [1]. Trans-phalangeal or -metatarsal amputations, collectively termed ‘minor amputations’ are an important component to prevent major limb loss and/or death. Resection of most, or all, of the necrotic or infected tissue is the first step towards attaining ulcer healing. Minor amputations for DFU are common and the incidence of DFUs across many settings is increasing [2].

The diagnosis and management of DFU infections and osteomyelitis is dependent on clinical signs and symptoms, in conjunction with blood laboratory, microbiological, and radiological evidence of infection. However, ascertaining and interpreting microbiological sample results is challenging [3]. Sources of microbiological sampling routinely collected in clinical practice include superficial wound swabs and deep tissue bone sample, either as a biopsy through intact skin, or through a surgical field [4]. For the latter, as part of a minor amputation procedure, it is commonly recommended that surgeons collect a sample of proximal bone (also known as ‘marginal bone’, or ‘bone chips’) for microbiological culture and, if possible, histopathological examination [5]. The rationale for intraoperative sampling is that the presence of infected bone, and thus residual osteomyelitis might prompt prolonged antibiotics or further surgery. Additionally, microbiological data from intraoperative bone sampling is thought to guide antibiotic choice [6].

Indeed, international guidelines recommend 2–5 days of antibiotic therapy following amputation with no residual infection. By contrast, for residual infected bone, a period of intravenous therapy is recommended, followed by oral antibiotics to complete 4–6 weeks treatment [6]. The data underpinning these recommendations are weak. Collecting proximal bone for culture is easier than histopathology, with a shorter turn-around time and without the need for expert pathologist examinations. The main caveat to culture in this context is that samples are collected through an infected surgical field, which makes interpretation of positive results difficult, particularly when these results may not accord with microbiological results collected from superficial swabs prior to the amputation [7].

The utility of obtaining a marginal bone specimen in determining which patients need further surgical or antibiotic management was identified as a knowledge gap in recent international guideline documents [5]. While superficial wound swabs are quick and easy to perform, it is regarded as an unreliable method of identifying the responsible pathogen, often thought to be inferior at identifying anaerobic or fastidious pathogens [8]. In the published literature, there are concerns regarding the degree of discordance between superficial swab and deep tissue microbiology, either collected by curettage or through unaffected skin [4, 8]. Whilst guidelines recommend against the routine collection of superficial wound swabs [5, 6], there are few data which explore the role of routine proximal bone culture as part of minor amputation procedures.

In this single centre retrospective study, we aimed to identify the degree of concordance between microbiological results of proximal bone culture collected routinely following minor amputation for infected DFU, in relation to results from superficial ulcer swabs and correlating with clinical outcomes in terms of complete healing, subsequent further operations, and major limb amputation. The purpose of this was to address the utility of routine acquisition of marginal bone specimens in determining which patients require further surgical or antimicrobial management. We hypothesised that the routine collection of marginal bone for culture has little clinical utility.

## Methods

Fiona Stanley Hospital (FSH) is a 783-bed tertiary hospital with a specialised multidisciplinary diabetes foot unit (MDFU). This multidisciplinary team comprises of endocrinologists, vascular surgeons, infectious diseases (ID) physicians, podiatrists, and community liaison nurses. The team manages complex diabetes-related foot complications across both inpatient and outpatient settings. A medical photographer attends all outpatient clinics to document ulcer site, size, and the presence of a healed wound. Outpatient wound care and, if required, parenteral antibiotics are provided by a single ambulatory nursing service.

The current study is a sub-study of the Audit of Multidisciplinary Diabetes Foot Unit services at Fiona Stanley and Fremantle Hospitals. Ethical approval was obtained from South Metropolitan Health Human Research Ethics Committee (RGS0000003204). To compare outcomes for patients hospitalised for diabetes related foot disease over time, two time periods of one year duration were studied in 2015 and 2019. For both the 2015 and 2019 cohorts this included hospitalisations during 52-week periods from the first week of February in each year. Between 2015 and 2019, clinical practice remained consistent, as endocrinology, ID and vascular surgery clinical leads remained the same.

The inclusion criteria for this study were as follows: 1] a diagnosis of diabetes, 2] admission for an infected diabetic foot ulcer and 3] minor amputation performed during admission. A minor amputation was defined as either a trans-phalangeal or trans-metatarsal amputation of single or multiple digits. Patients undergoing more complex surgeries including proximal forefoot amputations, revision of previous amputation sites or major lower extremity amputations were excluded.

In addition to the electronic medical record (EMR), data were also collected from laboratory and radiography databases. Demographics including age, sex, type of diabetes, glycaemic control, smoking history, diagnosis of chronic kidney disease and presence of Charcot neuroarthropathy were recorded. Glycaemic control was assessed with a pre-operative glycated haemoglobin (HbA1C). Smoking history was documented as never smoker or a history of previous/current smoking. Presence of chronic kidney disease (CKD) was judged based on the pre-operative eGFR (estimated glomerular filtration rate), and renal replacement therapy was noted. The history of an amputation was captured in the EMR and confirmed with review of operative notes. The requirement for an angioplasty during admission was also recorded.

Bone specimens were collected intraoperatively. Following bone transection, proximal bone samples were collected using a sterile ‘bone-nibbler’ and sent for culture. Our local standard of practice was for 2-weeks of oral antibiotics following the amputation, unless there was clinical evidence of residual soft tissue infection or involvement of adjacent osteoarticular structures. To define infection severity prior to the amputation, we applied the Infectious Diseases Society of America/International Working Group on the Diabetic Foot infection grading system (IDSA/IWGDF) [9, 10]. Amputations were recorded according to whether the most proximal amputation was trans-phalangeal or trans-metatarsal, whether they involved the first (hallux) or fifth ray, and the number of rays amputated. The microbiological results were obtained from superficial swabs (if collected within one month prior to admission) and proximal bone culture. Culture results were recorded according to bacterial species and if they were culture negative, monomicrobial or polymicrobial (> 1 named organisms reported by the microbiology laboratory).

We defined microbiology results as concordant if a swab and bone specimen were sent to the laboratory and the same organism(s) were isolated. We also considered concordant results if the bone cultures were negative, but an organism was isolated from superficial swabs. Pre-operative and post-operative antibiotic choice and planned duration was recorded. If discordance between swab and bone specimen was noted, the change in antibiotic choice was recorded.

Patient outcomes were assessed from the EMR up until six months post initial amputation. The primary outcome measure was the presence of complete healing by the end of the follow-up period. Secondary outcome measures included death, progression to a major amputation and the need for further surgery. If within this six-month period, a patient required intervention for a new ulceration within the immediate vicinity of the original amputation site, this was deemed contiguous with the original infection. Complete healing was defined as complete closure of the primary wound with no evidence of ulcer relapse within six months of the initial surgery. Further surgery was defined as further surgical intervention to the original amputation site, or a site within the immediate vicinity of the original infection.

Descriptive statistics were used to describe the study cohort using medians and interquartile range (IQR) for continuous variables and percentages for categorical variables. Mann Whitney U testing was performed for bivariate analyses of continuous variables, whilst a Chi-squared test was used for categorical variables. Logistic regression was performed with healing as the dependent variable. A backward stepwise approach was applied where explanatory variables with a *P*-value < 0.1 were eligible for inclusion and retained in the model if *P* < 0.05. The most parsimonious model was chosen on the basis of the Akaike Information Criterion. All statistical analyses were performed using R [11].

## Results

A total of 144 patients met the inclusion criteria and their characteristics are summarised (Table 1). The overall median (interquartile range [IQR]) age was 62 (53–73) years, and 97 (67.4%) patients were included from the 2019 cohort. Within the cohort, 114 (79.2%) individuals were male, and 133 (92.4%) had type 2 diabetes. The median (IQR) IDSA/IWGDF infection score was 3 [3, 4] and 48 (33.3%) individuals had trans-phalangeal amputation(s) while 96 (66.7%) patients had trans-metatarsal amputation(s). One quarter (25.7%) of the cohort had more than one ray amputated, and 24 (16.7%) patients had angioplasty performed during admission.

**Table 1:** Patient characteristics and concordance of data

*Patient characteristics*	All (144)	Swab and/or culture not done (33)	Concordant (73)	Discordant (38)	***P***-value
Age, years; median (IQR)	62 (53–73)	62 (53–73)	61 (52–72)	63 (55–74)	0.31
Sex: male, n; (%)	114 (79.2)	28 (84.8)	58 (79.4)	28 (73.7)	0.49
Type 2 Diabetes, n; (%)	133 (92.4)	33 (100)	64 (87.7)	36 (94.7)	0.24
Cohort recruited in 2019, n; (%)	97 (67.4)	24 (72.7)	52 (71.2)	21 (55.3)	0.53
HbA1c, %; median (IQR)	8.9 (7.6–10.9)	9 (7.8–11.1)	9 (7.6–10.9)	8.4 (7.4–9.8)	0.41
Chronic kidney disease stage, n; (%)					
0	71 (49.3)	19 (57.6)	8 (11.0)	17 (44.7)	0.74
1	2 (1.4)	0 (0)	2 (2.7)	0 (0)	
2	17 (11.8)	2 (6.1)	8 (11.0)	7 (18.4)	
3	33 (22.9)	6 (18.2)	18 24.7)	9 (23.7)	
4	15 (10.4)	5 (15.2)	6 (8.2)	4 (10.5)	
5	6 (3.5)	1 (3.0)	4 (5.5)	1 (2.6)	
Haemodialysis, n; (%)	3	1 (3.0)	1 (1.4)	1 (2.6)	0.64
Charcot neuroarthropathy (acute or chronic), n; (%)	10	3 (9.1)	3 (4.1)	4 (10.5)	0.19
IDSA/IWGDF Infection Score on admission	3 (3–4)	3 (3–4)	3 (3–3)	3 (3–3.75)	0.22
*Amputation characteristics*					
Amputation of first ray (hallux), n; (%)	47	11 (33.3)	25 (34.2)	11 (28.9)	0.57
Amputation of fifth ray, n; (%)	53	10 (30.3)	25 (34.2)	18 (47.4)	0.18
Most proximal amputation, n; (%)					
Transphalangeal	48 (33.3)	11 (33.3)	28 (38.4)	9 (23.7)	0.12
Transmetatarsal	96 (66.7)	22 (66.7)	45 (61.6)	29 (76.3)	
Amputation of > 1 ray, n; (%)	37 (25.7)	9 (27.3)	18 (24.7)	10 (26.3)	0.85
Angioplasty during admission, n; (%)	24 (16.7)	7 (21.2)	18 (24.7)	6 (15.8)	0.92
*Microbiology*					
Superficial swabs sent for culture	118 (81.9)	13 (39.4)	73 (100)	38 (100)	NA
Culture results from superficial swab, n (%)					
No growth	28 (19.4)	3 (23.1)	15 (20.5)	10 (26.3)	0.52
Monomicrobial	65 (45.1)	8 (65.5)	35 (47.9)	22 (57.9)	
Polymicrobial	25 (17.4)	2 (15.4)	17 (23.3)	6 (15.8)	
Time between swab and amputation, days; median (IQR)	3 (1–4)	NA	3 (1–4.5)	2 (1–5)	0.75
Marginal bone sample sent for culture, n; (%)	131 (91%)	20 (60.6)	73 (100)	38 (100)	NA
Culture results from marginal bone sample, n; (%)					
No growth	42 (29.2)	8 (40)	34 (46.6)	0 (0)	< 0.0001
Monomicrobial	51 (35.4)	4 (20)	30 (41.1)	17 (44.7)	
Polymicrobial	38 (36.4)	8 (40)	9 (12.3)	21 (55.3)	
Planned antibiotic duration post amputation, weeks; median (IQR)	2 (2–4)	2 (2–4)	2 (2–4)	2 (2–4)	0.61
*Outcome within 6 months*					
Complete healing, n; (%)	80 (61.5) [of 130]	13 (41.9) [of 31]	43 (67.1) [of 64]	24 (68.6) [of 35]	0.89
Further surgery, n; (%)	35 (26.7) [of 131]	12 (38.7) [of 31]	17 (26.6) [of 64]	6 (16.7) [of 36]	0.26
Major amputation, n; (%)	5 (3.8) [of 131]	2 (6.1)	3 (4.8) [of 63]	0 (0)	0.18
Death, n; (%)	4 (2.8) [of 143]	1 (3.0)	3 (4.2) [of 71]	0 (0)	0.20

*IQR* Interquartile range; *IDSA* Infectious Diseases Society of America; *IWGDF *International Working Group on the Diabetic Foot

Statistical analysis: Mann-Whitney U testing for continuous variables and Chi-squared testing for categorical variables

Both superficial wound swab and proximal bone culture results were available in 111 (77.1%) patients. Overall, culture of bone chips were more likely to yield coagulase negative staphylococcus (CoNS) (*P* < 0.05), Gram-negative organisms with chromosomally mediated inducible beta-lactamase activity (also known as ESCAPPM) [12] (*P* = 0.005), coliforms (*P* < 0.0001) and enterococcus (*P* < 0.0001) when compared with superficial wound swabs (Table 2). There was overall a moderate-high degree of concordance between microbiological samples, with only 38/111 (35%) of patients having discordant results between the superficial wound swab and proximal bone culture. Discordance was associated with polymicrobial bone culture results (*P* < 0.0001).

**Table 2:** Microbiological profiles from positive culture results from superficial swabs collected prior to surgery and marginal bone chips collected intra-operatively

	Swabs (90)	Bone chips (89)	*P*-value
Any Staphylococcus	60	45	0.17
MSSA	58	28	
MRSA	1	7	
S.lugdunensis	1	4	
CoNS	0	7	
Any beta-haemolytic streptococci	35	25	0.42
Group B	19	18	
* S. anginosus*	9	6	
* S. dysgalactiae*	4	1	
* S. pyogenes*	3	0	
Pseudomonas	6	3	0.30
ESCAPPM/Stenotrophomonas/ESBL	7	14	0.005
Coliforms	4	18	< 0.0001
Enterococcus	1	11	< 0.0001

*MSSA *methicillin-susceptible *Staphylococcus aureus*; *MRSA* methicillin-resistant *Staphylococcus aureus*; *CoNS* Coagulase negative Staphylococcus; *ESCAPPM* gram-negative organisms with chromosomally mediated inducible beta-lactamase activity; *ESBL* Extended spectrum beta lactamase

**Fig. 1:**
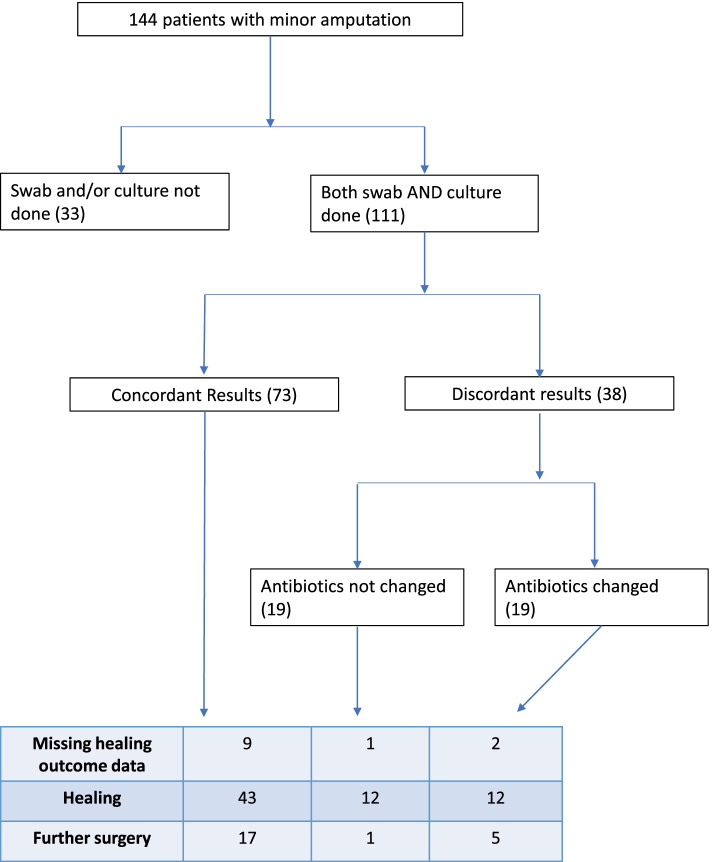
Outcomes associated with discordant microbiological results

Discordant microbiological results were not associated with adverse outcomes (Table 2). Complete healing was not associated with concordance. 67.2% of patients with concordant microbiology met the primary outcome compared to 68.6% of patients with discordant results (*P* = 0.89). Discordance was not associated with requiring further surgery. 26.6% patients with concordant results versus 16.7% individuals with discordant results required further surgery respectively (*P* = 0.26). With regards to major amputation and death rates, three patients with concordant results respectively went on to have a major amputation (*P* = 0.18) or passed away within the follow up period (*P* = 0.2). Of the 38 patients with discordant swab and bone chip results, half had a change in antibiotics, while the other half did not undergo a change to antibiotic therapy (Fig. 1). 12/18 (66.7%) individuals who did not undergo antibiotic changes met the primary outcome of healing, while 1/18 (5.6%) required further surgery. Of the individuals who underwent antibiotic change; 12/17 (70.5%) went on to meet the primary outcome, while 5/17 (29.4%) required further surgery.

Bivariate analyses of factors associated with healing are shown (Table 3). There was no difference in IDSA/IWGDF severity of infection grade between individuals who met the primary outcome versus those who did not. Age, sex, diabetes control, renal impairment, or presence of Charcot neuroarthropathy did not impact on healing. The type of amputation (trans-phalangeal/trans-metatarsal; *P* = 0.12), number of rays amputated (*P* = 0.43) and amputation of the first ray (*P* = 0.95) were not associated with healing. The most parsimonious logistic regression model demonstrated that revascularisation during admission (adjusted odds ratio (aOR); [95% confidence interval] 0.37 [0.13–0.96], *P* = 0.04) and amputation of the 5th ray (0.45 [0.21–0.94], *P* = 0.03) were independent risk factors for not healing.

**Table 3:** Clinical and microbiological factors associated with complete healing on bivariate analyses

***Patient characteristics***	All (144)	Healed at 6 months (80)	Not Healed (50)	***P***-value
Age, years; median (IQR)	62 (53–73)	60.5	62	0.33
Sex: male, n; (%)	114 (79.2)	62	44	0.49
Type 2 Diabetes, n; (%)	133 (92.4)	74	49	0.24
Cohort recruited in 2019, n; (%)	97 (67.4)	52/28	36/14	0.40
HbA1c, %; median (IQR)	8.9 (7.6–10.9)	8.7	9.1	0.44
Chronic kidney disease stage, n; (%)				
0	71 (49.3)	41	23	0.98
1	2 (1.4)	1	1	
2	17 (11.8)	10	6	
3	33 (22.9)	18	12	
4	15 (10.4)	7	5	
5	6 (3.5)	3	3	
Haemodialysis, n; (%)	3 (2.1)	1	2	0.31
Charcot neuroarthropathy (acute or chronic), n; (%)	10 (6.9)	6/74	4/46	0.91
IDSA/IWGDF Infection Score on admission (IQR)	3 (3–4)	3	3	0.64
*Amputation characteristics*				
Amputation of first ray (hallux), n; (%)	47 (32.6)	26	16	0.95
Amputation of fifth ray, n; (%)	53 (36.8)	26/54	25/25	0.05
Most proximal amputation, n; (%)				
Transphalangeal	48 (33.3)	30	13	0.12
Transmetatarsal	96 (66.7)	50	37	
Amputation of > 1 ray, n; (%)	37 (25.7)	19	15	0.43
Angioplasty during admission, n; (%)	24 (16.7)	9/71	12/38	0.05
*Microbiology*				
Superficial swabs sent for culture	118 (81.9)	70	35	0.01
Culture results from superficial swab, n (%)				
No growth	28 (19.4)	17	10	0.79
Monomicrobial	65 (45.1)	39	17	
Polymicrobial	25 (17.4)	14	8	
Time between swab and amputation, days; median (IQR)	3 (1–4)	3	3	0.98
Marginal bone sample sent for culture, n; (%)	131 (91%)	73	46	0.88
Culture results from marginal bone sample, n; (%)				
No growth	42 (29.2)	24	14	NS
Monomicrobial	51 (35.4)	28	18	
Polymicrobial	38 (36.4)	21	14	
Planned antibiotic duration post amputation, weeks; median (IQR)	2 (2–4)	2	3.5	0.06
*Outcome within 6 months*				
Further surgery, n; (%)	35 (26.7) of 131	8/71	24/24	< 0.0001

*IQR* Interquartile range; *IDSA* Infectious Diseases Society of America; *IWGDF* International Working Group on the Diabetic Foot

Statistical analysis: Mann-Whitney U testing for continuous variables and Chi-squared testing for categorical variables

## Discussion

Current guidelines do not encourage routine collection of superficial wound swabs in the management of infected DFUs [5, 6]. Certainly, there are concerns regarding the reliability of superficial wound cultures at accurately identifying the pathogenic organism, with documented discordance between deep tissue samples when collected as tissue curettage or as biopsies through unaffected skin [4, 8]. However, there is an argument suggesting that superficial wound swabs may offer complementary information, particularly in patients undergoing minor amputation. Some groups have demonstrated good correlation between superficial swab cultures and deep tissue samples; although these studies have been largely performed in smaller cohorts [13, 14]. Indeed, the results from our study supports this, having demonstrated a moderate-high degree of concordance between culture results obtained from superficial swabs and proximal bone samples. This questions the utility of routine intra-operative bone sampling.

Further to this, international guidelines currently recommend where possible, the use of intraoperative bone culture or histopathological sampling (when available) to accurately diagnose the presence of an infected DFU and osteomyelitis [5, 6]. The presence of residual infected bone implies residual osteomyelitis with guidelines recommending prolonged antibiotics or further surgery [5, 6]. Our results, however, demonstrate no statistical difference in healing outcomes, need for further surgery, or progression to major amputation and death amongst the small proportion of patients who had discordant microbiological sample results, regardless of antibiotic changes. As such, the presence of positive microbiological bone samples did not necessarily correlate with worse outcomes for our cohort. These results are contrary to results from other groups who identified higher rates of poor outcomes in patients with positive marginal bone samples; although we acknowledge their sample sizes were small [15]. Similar to these groups, in our cohort, the extent of surgery was made at the discretion of the treating vascular surgeon based on the severity of local and systemic infection. The choice and duration of antibiotics was made using Australian Therapeutic Guidelines recommendations and tailored according to the patient’s current and previous culture results at the discretion of the ID physicians [16]. Most patients received empiric antibiotics pre-operatively.

Where discordant results were obtained, intra-operative bone samples were more likely to be polymicrobial with higher proportions of CoNS, ESCAPPM, coliforms and enterococci. These findings are likely to be due to differences in how the laboratory handles swabs and tissue specimens. All organisms identified from bone specimens are formally identified to the species level, and susceptibility profiles reported. Notwithstanding these handling differences, these findings are likely related to the risk of intra-operative sample contamination during collection [7].

Unsurprisingly, these results demonstrated that the need for an inpatient angiogram had a higher association with non-healing. Ischaemia related to peripheral arterial disease is a well-known risk factor for non-healing ulcers, infection, and further amputation [17]. If a patient required an angiogram during the admission, it likely suggests the presence of severe or critical limb ischaemia which impair healing. Additionally, a 5th ray amputation was another independent risk factor for a non-healing amputation site. Infection and ulceration along the lateral column of the foot are often severe and complicated [18, 19]. While necessary, amputation of the 5th ray often results in poor wound healing and recurrent wounds due to changes in foot structure and function [18, 19].

This study has limitations. The sample size was relatively small, and due to its retrospective nature, relied on the contents of the EMR. As such, outcomes of some patients had to be excluded due to loss to follow-up or absence of documentation in the EMR. Histopathology was not performed on any patient, which limits any conclusion on the utility for this modality in defining patients at risk for poor outcomes. While histopathological assessment of bone samples may be useful in correlating microbiology from swab and bone samples to further ascertain the utility of intra-operative bone sampling, its practicality and reliability needs to assessed in a future study.

## Conclusion

This study demonstrates a moderate-high degree of concordance between superficial wound swab and intra-operative bone sample microbiology. Discordance between the microbiological samples did not result in significantly worse outcomes and this was independent of changes made to the antibiotic choice following identification of discordance suggesting little clinical utility in routinely collecting proximal bone for culture during minor amputation.

As a result, choice and duration of antibiotic therapy may be determined by superficial wound swab cultures and clinical correlation with evidence of residual soft tissue infection or osteoarticular involvement of adjacent structures. Further research should evaluate the clinical utility, reliability, and cost-effectiveness of such an approach in combination with histopathological assessment.

## Data Availability

All relevant data analysed during this study is contained within this article.

## Abbreviations

DFU: Diabetes-related foot ulcers
FSH: Fiona Stanley Hospital
MDFU: Multidisciplinary diabetes foot unit
ID: Infectious diseases
EMR: Electronic medical record
HbA1C: Glycated haemoglobin
CKD: Chronic kidney disease
eGFR: Estimated glomerular filtration rate
IDSA: Infectious Diseases Society of America
IWGDF: International Working Group on the Diabetic Foot
IQR: Interquartile range
CoNS: Coagulase negative staphylococcus
ESCAPPM: Gram-negative organisms with chromosomally mediated inducible beta-lactamase activity
aOR: Adjusted odds ratio
